# Rubinstein-Taybi syndrome in a Saudi boy with distinct features and variants in both the *CREBBP* and *EP300* genes: a case report

**DOI:** 10.1186/s12881-019-0747-5

**Published:** 2019-01-11

**Authors:** Mohammad M. Al-Qattan, Abdulaziz Jarman, Atif Rafique, Zuhair N. Al-Hassnan, Heba M. Al-Qattan

**Affiliations:** 1 Division of Plastic Surgery, King Saud University, PO Box 18097, Riyadh, 11415 Saudi Arabia; 2 Division of Plastic Surgery, King Faisal Specialist Hospital and Research Center, PO Box 18097, Riyadh, 11415 Saudi Arabia; 3 Division of Plastic Surgery, King Abdulaziz Medical City, Ministry of National Guard Health Affairs, PO Box 18097, Riyadh, 11415 Saudi Arabia; 4 Department of Medical Genetics, King Faisal Specialist Hospital and Research Center, Riyadh, Saudi Arabia

**Keywords:** Rubinstein-Taybi syndrome, *CREBBP*, *EP300*

## Abstract

**Background:**

Rubinstein-Taybi syndrome (RSTS) Type 1 (OMIM 180849) is characterized by three main features: intellectual disability; broad and frequently angulated thumbs and halluces; and characteristic facial dysmorphism.

**Case presentation:**

We report on a Saudi boy with RSTS Type 1 and the following distinct features: a midline notch of the upper lip, a bifid tip of the tongue, a midline groove of the lower lip, plump fingers with broad / flat fingertips, and brachydactyly. The child was found to be heterozygous in the *CREBBP* gene for a sequence variant designated c.4963del, which is predicted to result in premature protein termination p.Leu1655Cysfs*89. The child and his father were also found to be heterozygous in the *EP300* gene for a sequence variant designated c.586A > G, which is predicted to result in the amino-acid substitution p.Ile196Val.

**Conclusion:**

Our report expands the clinical spectrum of RSTS to include several distinct facial and limb features. The variant of the *CREBBP* gene is known to be causative of RSTS Type 1. The variant in the *EP300* gene is benign since the father carried the same variant and exhibited no abnormalities. However, functional studies are required to investigate if this benign *EP300* variant influences the phenotype in the presence of disease-causing *CREBBP* gene mutations.

## Background

There are two types of Rubinstein-Taybi syndrome (RSTS): Type 1, which is caused by mutations of the *CREBBP* gene; and Type 2, which is caused by mutations of the *EP300* gene. Both types are autosomal dominant. Clinically, the mutations are almost always seen as de novo [1].

RSTS Type 1 (OMIM 180849) is characterized by three main features: intellectual disability; broad and frequently angulated thumbs and halluces; and characteristic facial dysmorphism, namely highly-arched eyebrows, down-slanting palpebral fissures, a broad nasal bridge, a columella hanging below the *alae nasi*; a thin upper lip, pouting of the lower lip, and mild micrognathia. Several other variable features have also been described, such as cardiac defects, a highly-arched palate with or without a bifid uvula, low-set posteriorly-rotated ears, patellar dislocation, reduced immunity, and undescended testes. Furthermore, patients have an increased risk of developing tumors, especially those of the nervous system [1–3]. RSTS Type 2 (OMIM 613684) has features similar to those of Type 1, although the features tend to be milder. Nonetheless, each type exhibits specific clinical features [1–13]; and these genotype-phenotype correlations are summarized in Table 1.

**Table 1:** Differences in the phenotypes of RSTS Type 1 (Caused by *CREBBP* mutations) and Type 2 (caused by *EP300* mutations)

Clinical Features	Differences in the phenotypes
Facial dysmorphism	- Both types have the following classic features [1–12]: highly- arched eyebrows, long eye lashes, broad nasal bridge, over-hanging columella, thin upper lip, pouting lower lip, posteriorly rotated ears, and micrognathia. These features tend to be milder in almost all cases of Type 2, and also in cases of Type 1 caused by missense mutations of the *CREBBP* gene [2, 11]. - The following features are common in Type 1 and are uncommon in Type 2: down-slanting of the palpebral fissures, and a grimacing smile [1, 12]. - Helical pits are occasionally seen in type 2 [12].
Intra-oral features	- Talon cusps (an accessory cusp-like structure on the lingual side of the permanent incisors, resembling the shape of an eagle’s talon), and a highly-arched palate are common in both types. Bifid uvula and bifid tip of the tongue are rare features [8].
Mental/learning defects	- intellectual disability is milder in Type 2 [2]. Learning disability without intellectual disability is frequently seen in Type 2 [12].
The hands/feet	- Bilateral broadening of the thumbs/halluces is a feature of both types. However, the angulation of the thumbs/halluces are much less frequent in Type 2 [9]. Normal thumbs/halluces may be seen in Type 2 [10]. - The following occasional features are characteristic of Type 2: short first metatarsals, fetal finger tip pads (prominence of the ventral aspects of the finger tips), overlapping toes, syndactyly, and brachydactyly of the 5th toes [1, 4–6].
Other features	- Seizures are more common in Type 1 [10] - Microcephaly [9] and hirsutism [6] are more common in Type 2. - Cardiac defects may be seen in both types. - The following occasional features are characteristic of Type 1: patellar dislocation, reduced immunity (usually presenting as recurrent respiratory tract infections), undescended testes, and an increased risk of tumors of the nervous system [1–3, 11, 13]. - The following occasional features are characteristic of Type 2: scoliosis and swallowing difficulties [4, 5].
Preeclampsia of the mother during pregnancy	- Seen in 23% of mothers of *EP300* mutated patients compared to only 3% of mothers of *CREBBP* mutated patients [9].

In this paper, we report on a Saudi boy with RSTS Type 1 and the following distinct features: a midline notch of the upper lip, a bifid tip of the tongue, a midline groove of the lower lip, plump fingers with broad / flat fingertips, and brachydactyly.

## Case presentation

**Fig. 1:**
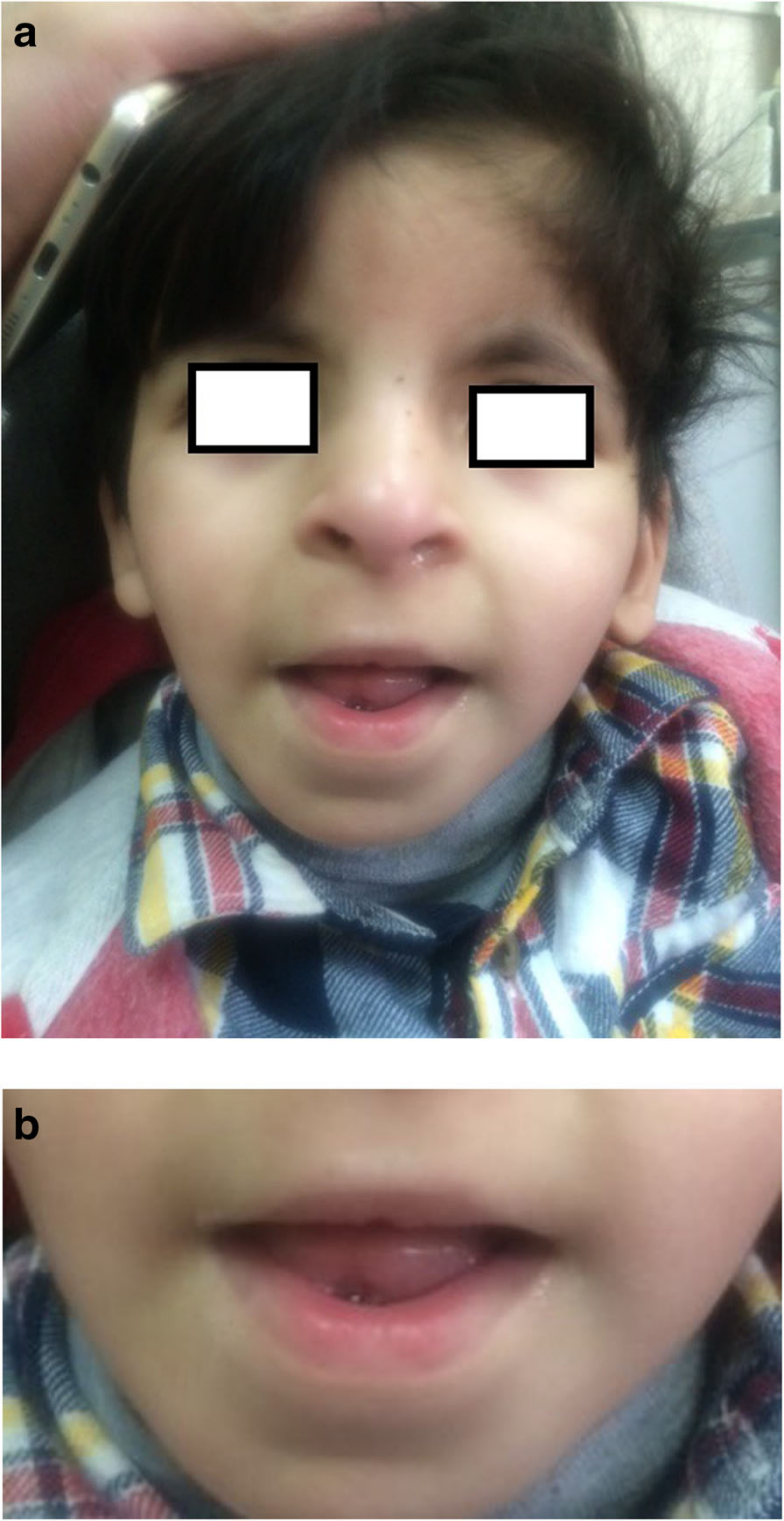
**a** Facial features of the patient. Classic features present are: highly-arched eyebrows, down-slanted palpebral fissures, a broad nasal bridge, a columella hanging below the *alae nasi*, low-set posteriorly-rotated ears, a thin upper lip, pouting of the lower lip, and mild micrognathia. Unique facial features are: a midline notch of the upper lip, a bifid tip of the tongue, and a midline groove of the lower lip. **b** A close-up view showing the unique facial features

**Fig. 2:**
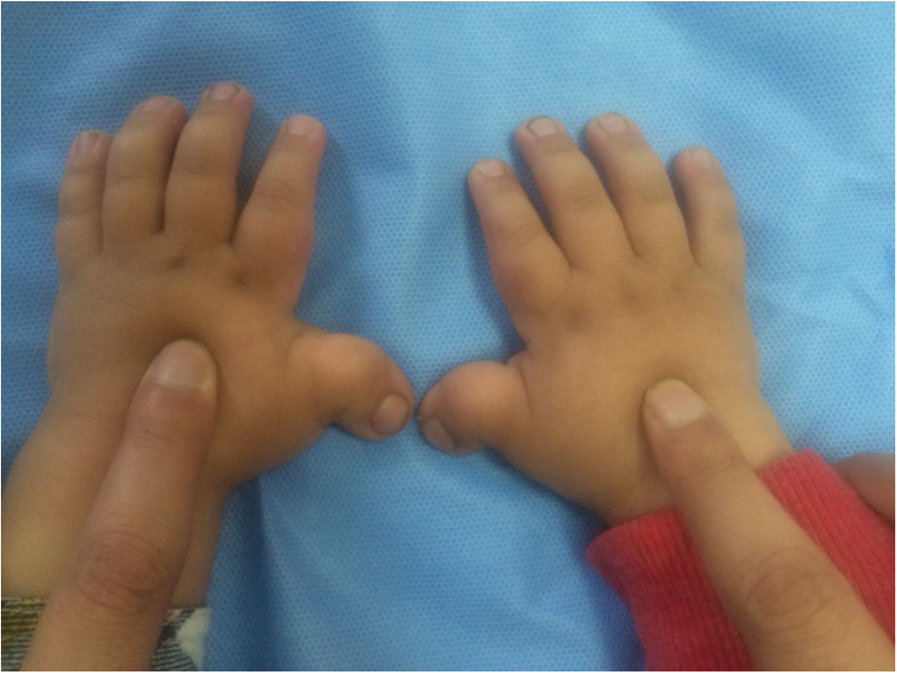
Clinical appearance of the hands. Note the classic feature of broad, flat, angulated thumbs. The unique features are the plump fingers with broad/flat fingertips, as well as the brachydactyly of the fingers

**Fig. 3:**
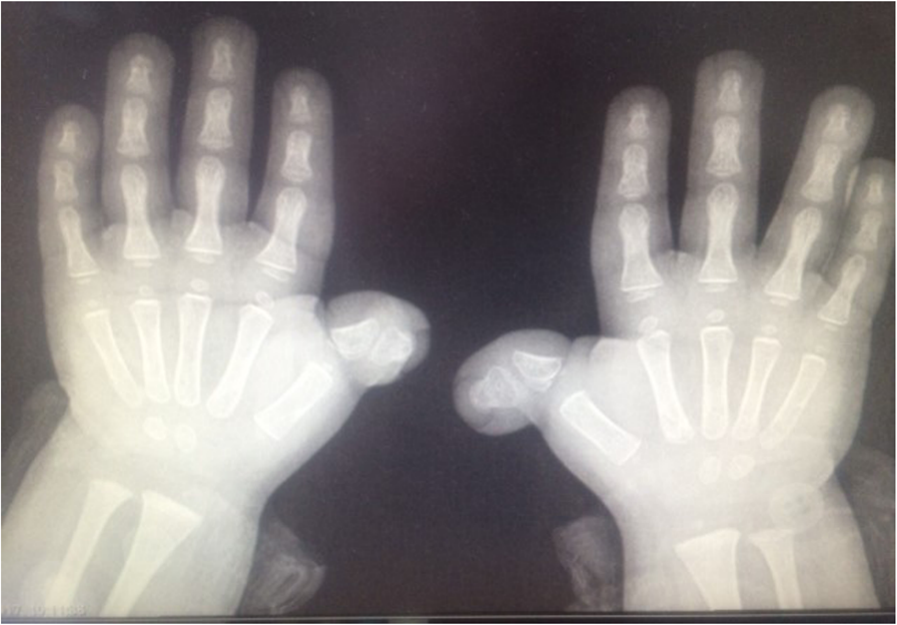
X-rays of the hands. The angulated thumbs are caused by a delta-phalanx

**Fig. 4:**
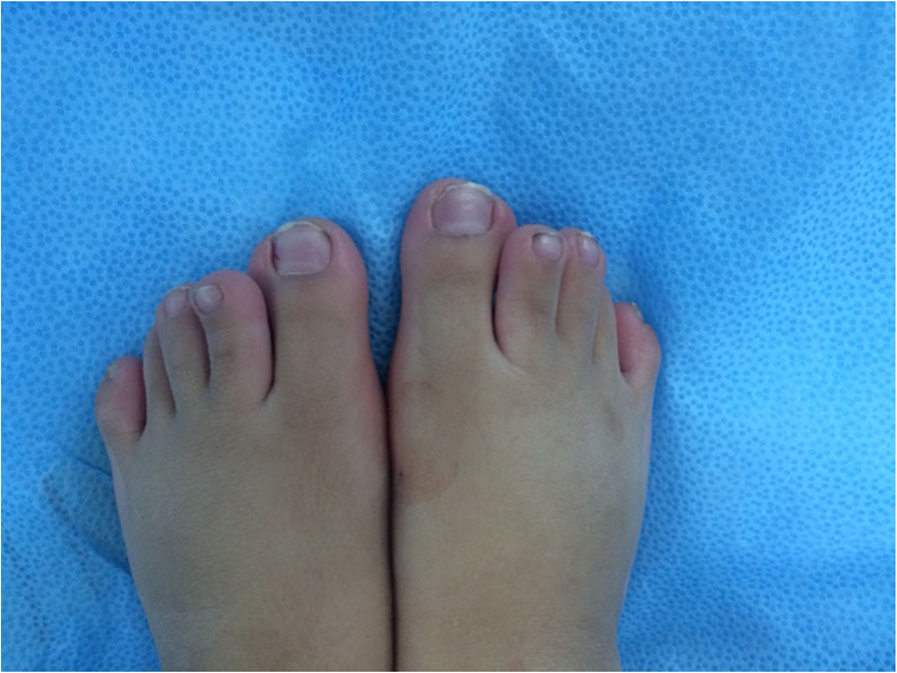
Clinical appearance of the feet. The big toes are broad, and the remaining toes overlap. Note the brachydactyly of the second toes

A 4-year old Saudi boy presented to the Hand Clinic for surgical correction of his angulated thumbs. He was an only child of non-consanguineous parents. He was born vaginally at term after an uneventful pregnancy. The birth weight and length were at the 30th centile. Family history was unremarkable. The patient had all the hallmark features of RSTS Type 1 including: intellectual disability, typical facial dysmorphism (highly-arched eyebrows, down-slanted palpebral fissures, a broad nasal bridge, a columella hanging below the *alae nasi*, low-set posteriorly-rotated ears, a thin upper lip, pouting of the lower lip, a highly-arched palate, and mild micrognathia), broad / flat/ angulated thumbs, broad big toes, and overlapping post-axial toes (Figs. 1, 2, 3 and 4). The child also had the following distinct features: a midline notch of the upper lip, a bifid tip of the tongue, a midline groove of the lower lip, plump fingers with broad / flat fingertips, and brachydactyly (Figs. 1b, 2, 4). The child had a history of cardiac surgery (correction of an atrial septal defect and repair of hemi-anomalous pulmonary venous drainage) as well as orchiopexy (for an undescended left testis). Full systemic examination and radiological investigations did not reveal any other defects. Both parents had no abnormalities.

After informed consent was obtained, genomic DNA was extracted from the peripheral blood of the child and both parents. For testing, a combination of Next Generation Sequencing (NGS) and Sanger Sequencing was used to cover the full coding regions of the tested genes plus 20 bases of the non-coding DNA flanking each exon. The most important two syndromes with features overlapping with those of RSTS are the Cornelia de Lang (OMIM12247) and Floating-Harbor (OMIM 136140) syndromes [14, 15]. Hence, all the genes known to cause RSTS, Cornelia de Lang syndrome, and the Floating-Harbor syndrome (see Table 2) were sequenced. The sequences were then aligned and compared with reference sequences. All differences from the reference sequences (sequence variants) were assigned to one of the five interpretation categories as per ACMG guidelines [16].

**Table 2:** Genes sequenced and their transcript numbers

Genes sequenced	Transcript numbers	Syndrome
*CREBBP*	NM_004380.2	Rubinstein-Taybi
*EP300*	NM_001429.3	Rubinstein-Taybi
*NIPBL*	NM_133433.3	Cornelia de Lang
*SMC1A*	NM_006306.3	Cornelia de Lang
*SMC3*	NM_005445.3	Cornelia de Lang
*RAD21*	NM_006265.2	Cornelia de Lang
*HDAC8*	NM_018486.2	Cornelia de Lang
*SRCAP*	NM_006662.2	Floating-Harbor

**Fig. 5:**
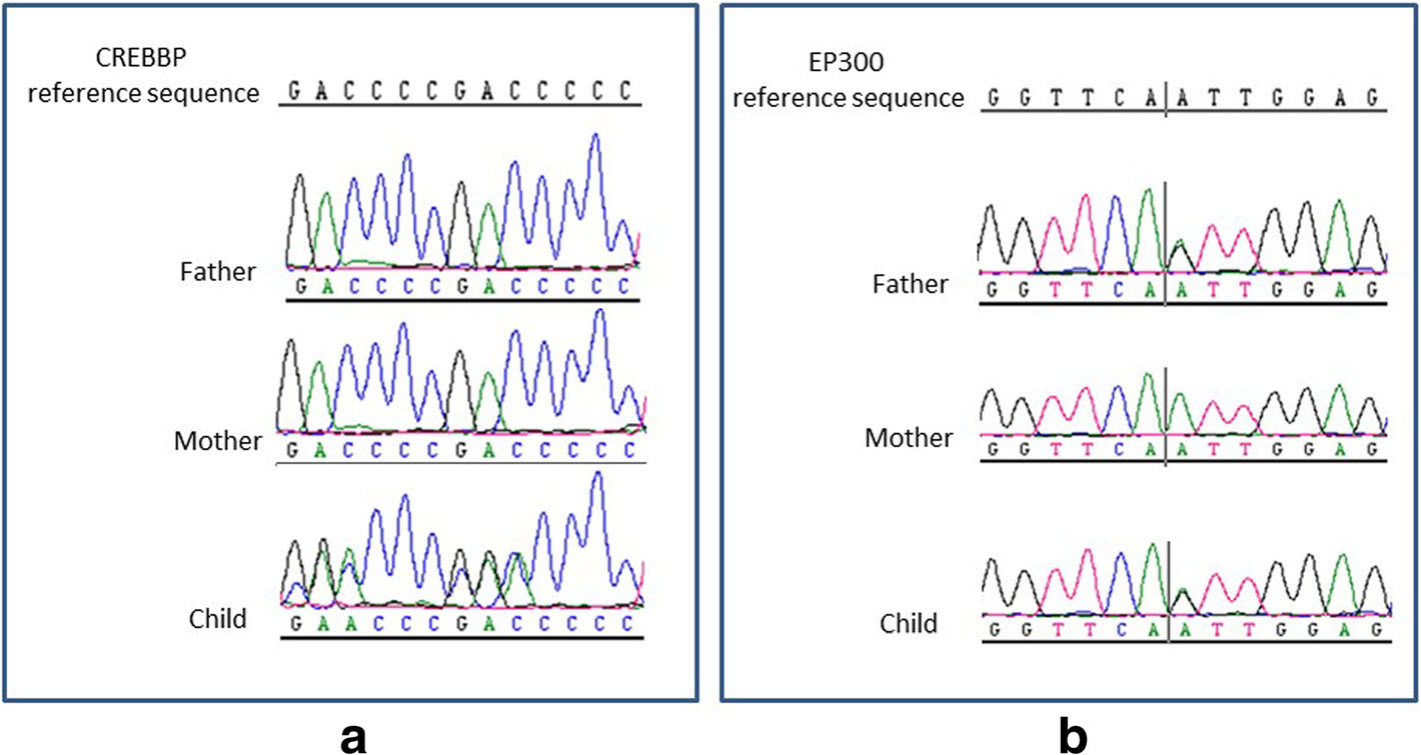
**a** Sanger sequencing results of the variant in *CREBBP*. The child is heterozygous for the variant, but it was not detected in the parents. **b** Sanger sequencing results of the variant in *EP300*. The child and his father are heterozygous for the variant, but it was not detected in the mother

The child was found to be heterozygous in the *CREBBP* gene for a sequence variant designated c.4963del, which is predicted to result in premature protein termination p.Leu1655Cysfs*89. This variant has been reported to be causative of RSTS Type 1 [7]. The variant was not detected in the parents (Fig. 5a), which indicates that it is a de novo event. According to the criteria of the ACMG, this variant is classified as pathogenic.

The child and his father were also found to be heterozygous in the *EP300* gene for a sequence variant designated c.586A > G, which is predicted to result in the amino-acid substitution p.Ile196Val (Fig. 5b). This variant appears to be rare in large population databases of genetic variations (http://exac.broadinstitute.org/variant/22-41513682-A-G). The amino-acid residue p.Ile196 of the EP300 protein has been highly conserved during evolution. The variant is predicted by SIFT, Polyphen-2, and Mutation Taster to be benign. According to Clinvar, the variant has been designated of uncertain significance by one submitter and likely benign by another submitter (https://www.ncbi.nlm.nih.gov/clinvar/variation/158567/). According to the criteria of the ACMG, this variant is classified as likely benign.

## Discussion and conclusions

Our report expands the clinical spectrum of RSTS to include several distinct facial and limb features.

Three unique oral features were seen in the midline of our patient. The midline notch of the upper lip may be considered a microform median cleft of the upper lip. Patients with RSTS frequently have a highly arched palate and occasionally a bifid uvula. However, a median upper lip cleft has not been previously described [8]. Similarly, the median groove of the lower lip has not been previously described in RSTS. However, bifidity of the tip of the tongue has been previously described, but is a rare feature in RSTS [8].

The other distinct features seen in our patient were plump fingers with broad / flat fingertips, as well as brachydactyly of all the fingers and the second toes. Our review of the literature did not reveal any reported case of RSTS with these features. It should be noted that RSTS patients frequently present with brachydactyly of the distal phalanx of the thumbs (also known as brachy-telephalangism) [2]. Furthermore, short first metatarsals and brachydactyly of the 5th toes have been described in RSTS Type 2 (see Table 1) [1].

The most important two syndromes with overlapping features with RSTS are the Cornelia de Lang and Floating-Harbor syndromes [14, 15]. All 3 syndromes share many of the facial features as well as the intellectual disability. Characteristic features of Cornelia de Lang syndrome include: synophrys (the two eyebrows meeting in the middle above the bridge of the nose), common occurrence of ophthalmic features (such as nystagmus and myopia), gastrointestinal reflux, and hearing loss. Characteristic features of the Floating-Harbor syndrome include proportionate short stature, delayed speech development, and delayed bone age. However, it is frequently difficult to differentiate between the Floating-Harbor syndrome and RSTS clinically. This is not surprising since the causative gene of the former syndrome (the *SRCAP* gene) encodes the SNF2-related CREBBP activator protein [15]. Our patient did not have any of the characteristic features of the Cornelia de Lang and Floating-Harbor syndromes, and the patient tested wild-type to their causative genes (See Table 2).

Woods et al. [17] reported on a patient with RSTS Type 2 and a novel *EP300* frame shift mutation with features that overlap those of the Cornelia de Lange syndrome (OMIM 122470) including the characteristic synophrys. This was thought to be important for the clinician since intellectual disability, arching of the eyebrows, and hirsutism are features of both RSTS and Cornelia de Lang syndromes. Similarly, a microform median upper lip cleft, a bifid tongue, and brachydactyly are characteristic features of oro-facio-digital syndrome Type 1 (OFD1, OMIM 311200) [18]. Hence, our case shows that patients with RSTS Type 1 may have features that overlap with those of OFD1. However, OFD1 patients have several other differentiating facial and systemic features such as sparse hair, alopecia, milia, lingual hamartomas, cerebral/cerebellar abnormalities, and polycystic kidneys. Knowledge of these overlapping features is also important for clinical identification, especially in cases of RSTS with a mild phenotype (also known as the incomplete RSTS) [19].

The truncating mutation in the *CREBBP* gene of our patient was within the HAT (Histone Acetyl Transferase) domain. Most previously reported truncating and missense mutations of the *CREBBP* gene were clustered within the HAT domain [7]. The HAT domain promotes histone acetylation which affects the transcriptional availability of chromatin [10].

Another interesting finding in our patient was the presence of the benign variant in the *EP300* gene. The father carried the same variant and exhibited no abnormalities. However, functional studies are required to investigate whether this variant affects the phenotype in the presence of *CREBBP* gene mutations. This is theoretically possible because the encoded proteins of the two genes have homologous binding sites for several transcription factors [20].

## Abbreviations

ACMG: American College of Medical Genetics and Genomics
CREBBP: Cyclic AMP- Response Elements-Binding Protein
EP300: E1A-Binding Protein 300
HAT: Histone acetyl transferase
NGS: Next generation sequencing
OFD1: Oral-facial digital syndrome Type 1
RSTS: Rubinstein-Taybi syndrome
SIFT: Sorting intolerant from tolerant
SNF2: SWI/SNF-related matrix-associated actin-dependent regulator of chromatin
